# Cyclic Relaxation, Impact Properties and Fracture Toughness of Carbon and Glass Fiber Reinforced Composite Laminates

**DOI:** 10.3390/ma14237412

**Published:** 2021-12-03

**Authors:** Mohammed Y. Abdellah, Mohamed K. Hassan, Ahmed F. Mohamed, Ahmed H. Backar

**Affiliations:** 1 Mechanical Engineering Department, College of Engineering and Islamic Architecture, Umm Al-Qura University, Makkah 21955, Saudi Arabia; mkibrahiem@uqu.edu.sa (M.K.H.); afmohamed@uqu.edu.sa (A.F.M.); ahbackar@uqu.edu.sa (A.H.B.); 2 Mechanical Engineering Department, Faculty of Engineering, South Valley University, Qena 83521, Egypt; 3 Production Engineering& Design Department, Faculty of Engineering, Minia University, Minia 61111, Egypt; 4 Mechanical Engineering Department, Faculty of Engineering, Sohag University, Sohag 82524, Egypt; 5 Production Engineering Department, Faculty of Engineering, Alexandria University, Alexandria 21544, Egypt

**Keywords:** relaxation, drop weight, cyclic, fiber, composites

## Abstract

In this paper, the mechanical properties of fiber-reinforced epoxy laminates are experimentally tested. The relaxation behavior of carbon and glass fiber composite laminates is investigated at room temperature. In addition, the impact strength under drop-weight loading is measured. The hand lay-up technique is used to fabricate composite laminates with woven 8-ply carbon and glass fiber reinforced epoxy. Tensile tests, cyclic relaxation tests and drop weight impacts are carried out on the carbon and glass fiber-reinforced epoxy laminates. The surface release energy G_IC_ and the related fracture toughness K_IC_ are important characteristic properties and are therefore measured experimentally using a standard test on centre-cracked specimens. The results show that carbon fiber-reinforced epoxy laminates with high tensile strength give high cyclic relaxation performance, better than the specimens with glass fiber composite laminates. This is due to the higher strength and stiffness of carbon fiber-reinforced epoxy with 600 MPa compared to glass fiber-reinforced epoxy with 200 MPa. While glass fibers show better impact behavior than carbon fibers at impact energies between 1.9 and 2.7 J, this is due to the large amount of epoxy resin in the case of glass fiber composite laminates, while the impact behavior is different at impact energies between 2.7 and 3.4 J. The fracture toughness K_IC_ is measured to be 192 and 31 MPa √m and the surface energy G_IC_ is measured to be 540.6 and 31.1 kJ/m^2^ for carbon and glass fiber-reinforced epoxy laminates, respectively.

## 1. Introduction

Fiber-reinforced plastics (FRP) are used in many infrastructure and aerospace applications. These composites are usually used as laminates, which have higher specific strength and are lighter than standard metals. In terms of fracture behavior, these composites are classified as quasi-brittle materials. In the presence of holes and enlarged geometries, their behavior lies somewhere between brittle and ductile [1,2,3,4,5,6]. The mechanical properties of glass fiber-reinforced polymer (GFRP) structures were studied in [1,2,3,4] to understand the size effect produced by the hole under static loading. The mechanical behavior under impact loading was studied by Abdellah et al. [7]. The addition of a steel mesh between the fiberglass layers in a composite laminate increases ductility while decreasing fracture toughness and tensile strength and improving damage tolerance. Moreover, the steel mesh minimizes the size effect of the composite plate with an open circular hole [8]. The vibration behavior of such a composite was investigated [8]. The fracture toughness of glass fiber composites has been measured experimentally in many studies [2,9,10]. A work by Abdellah et al. [7] studied the impact and relaxation loading of glass fiber-reinforced composite laminates. Fouad et al. [9] measured the fracture toughness of epoxy resin reinforced with carbon fibers, Kevlar and glass fibers for biomedical applications. Delamination was considered as a damage type observed in composite laminates [11,12]. The study of delamination due to impact [12,13] has been strongly recommended to fully understand its behavior, especially in materials such as laminates that are considered to have low impact resistance and damage tolerance.

The basic concepts of the viscoelastic behavior of polymer composites under relaxation and creep were described by Papanicolaou and Zaoutsos [14]. Composites are considered as viscoelastic materials affected by fatigue [15] and even by creep [16]. The effect of short elastic fibers on the relaxation behavior of composites was studied analytically [17] and evaluated numerically using a Monte Carlo finite element analysis. It was found that stress relaxation is affected by the elastic and shear moduli of the matrix. George et al. [18] investigated a chemical surface treatment of fibers, which increased the interfacial bonding between the fiber and the matrix and resulted in a decrease in relaxation stress. The same chemical treatment results were observed by Pothan et al. [19]. An analytical study by Obaid et al. [20] was carried out on composites reinforced with short and randomly oriented fibers to understand the behavior and interfacial bonding during stress relaxation. A finite element model by Obaid et al. [21] provided a good description and explanation of the change in stress relaxation constant with the interfacial shear stress at the fiber-matrix interfaces. Further work reported that the chemical interaction between the fiber and the matrix largely affects the stress relaxation [22,23]. This interfacial reaction depends on the molecular structure [24,25,26]. In addition to the effect of chemical treatment at the fiber-matrix interfaces on stress relaxation, the effect of binder, degree of elongation, fiber loading, fiber orientation, and temperature have also been studied in short sisal fiber-reinforced natural rubber [27]. Saeed et al. [21] studied stress relaxation at the interfaces of glass fiber-reinforced high density polyethylene (HDPE). These results were compared with Cox’s analytical model of shear retardation. A study by Fathy et al. [28] investigated the stress relaxation behavior of composite materials used in underground pipelines.

As previously reported, much work has been done to investigate relaxation stress in relation to chemical treatment and interfacial debonding at fiber-matrix interfaces. The relaxation stresses are important to fully understand the behavior under different strain and loading conditions, as they help in predicting the dimensional stability of the load bearing capacity of composites and in determining the loading by modulus for composite-bolted joints [29]. Moreover, the stress relaxation study is important for the curing process of composite structures where dimensional stability over time is needed. Therefore, the present work has two objectives: (1) to study the effect of stress increases (central open holes) on stress relaxation and (2) to understand delamination damage during a drop-weight impact test. These properties require a complete description of the fracture toughness and tensile properties of glass- and carbon fiber-reinforced polymers (GFRP and CFRP).

The article is organized as follows: In the first part, the concepts of stress relaxation are explained; in the second part, the fabrication of GFRP and CFRP is discussed; in the third part, the tensile, notched bar impact, relaxation and drop weight tests are explained. In the last section, the results and discussion are presented.

## 2. Stress Relaxation

**Figure 1:**
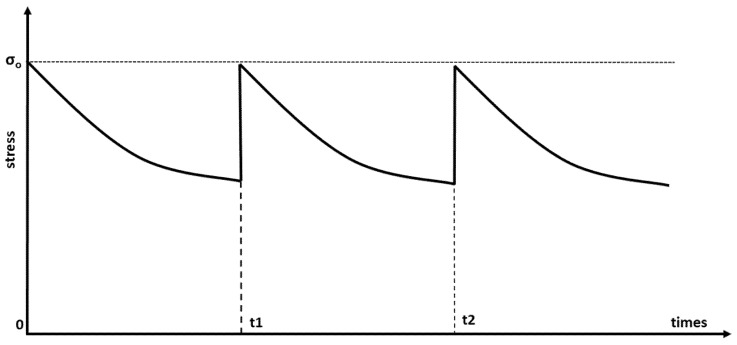
Cyclic stress versus time relation [30].

The applied stress is kept constant throughout the stress relaxation test, and the change in stress over time is observed. Under continuous stress, the stress level gradually decreases. At temperatures above Tg, the stress relaxation is significant, while at temperatures below Tg, it is negligible. For this reason, the stress relaxation test is performed at temperatures above and below Tg. The temperature in the chamber is kept at a constant level throughout the test. The stress relaxation test is performed under compression in the case of shape memory polymer foam. The test is performed using a tensile or compression testing machine when short term properties are being investigated. For the investigation of long-term properties, the test is performed with a constant displacement machine in a chamber with temperature control. Stress relaxation is automatically recorded during the test. In the stress relaxation test, the change in stress under constant strain e0 with time is recorded. The stress relaxation is monitored until time t1 is reached. In a cyclic stress relaxation test, the stress s0 is determined by applying strain e1 at time t1 and the stress response after t1 is recorded. These loading processes are repeated in a cyclic test, as shown in Figure 1 [30].

**Figure 2:**
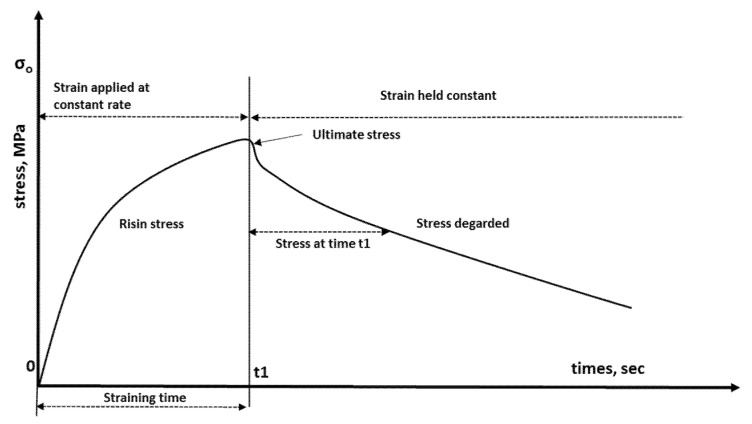
Schematic drawing of a stress–time relationship.

Under constant loading conditions, stress relaxation is a time-dependent decrease in stress. By applying a specific deformation to a specimen and measuring the stress required to maintain that deformation as a function of time, this particular polymer behavior can be studied. Stress relaxation data have proven valuable in a variety of practical applications. Figure 2 shows a typical stress–time curve. To achieve the desired strain, a uniform strain rate was applied to the sample at the beginning of the experiment. Once the sample reached the desired strain, the strain was held constant for a specified time. As a function of time, the stress drop that occurs due to stress relaxation is noted. The stress measurements are recorded at different time intervals and the results are plotted as a graph of stress versus time.

## 3. Materials and Methods 

### 3.1. Hand Layup

**Figure 3:**
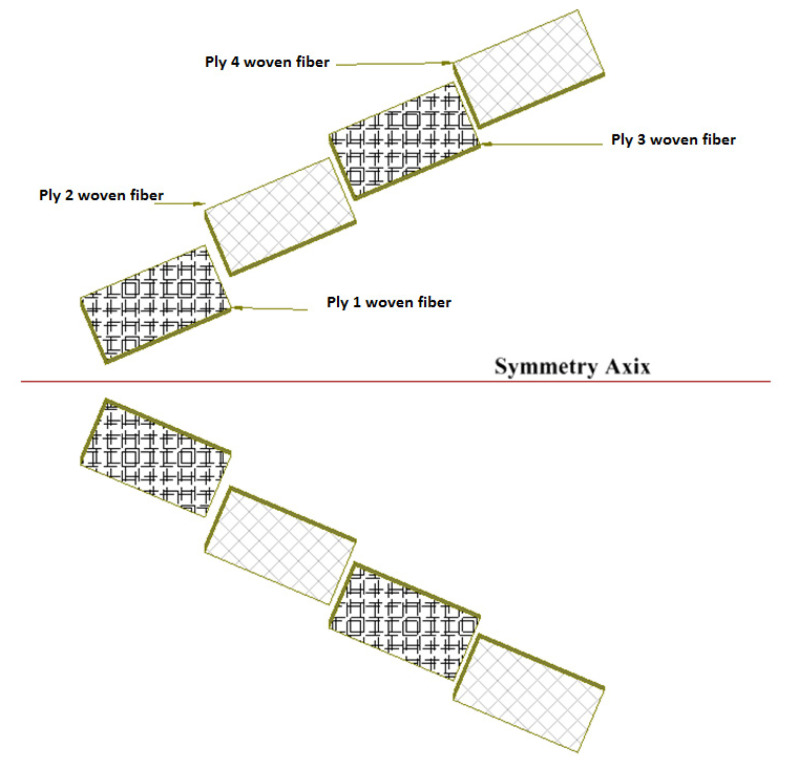
A schematic of the different plies used in manufacturing S2.

Laminated composite structures are manufactured using a variety of complicated production methods. Therefore, the hand lay-up method [31,32,33,34,35,36], which is considered the cheapest and simplest, was recommended and chosen. In the fabrication of this method, processes with two-sided glass plates are used. One plate serves as a base and is waxed with a release agent to prevent sticking. Then, a single layer of epoxy resin (see Table 1 [32,37,38] for mechanical properties) is applied evenly to the entire base plate. Then, glass fibers are placed on top of the epoxy resin layer to build up another layer, and the process is repeated until all laminate layers are formed and completed according to the build-up sequence shown in Figure 3. As shown in Figure 3, each laminate has eight layers of woven glass fibers (S1) and eight layers (S2) of woven carbon fibers. The ASTM D3171-99 standard [39] allows the use of the ignition removal approach. The average thickness of the fabricated sheets for samples S1 and S2 was 3.4 mm and 1.7 mm, respectively. The volume fractions of the fibers were measured to be 65% for CFRP and 45% for GFRP.

### 3.2. Tensile Test

**Figure 4:**
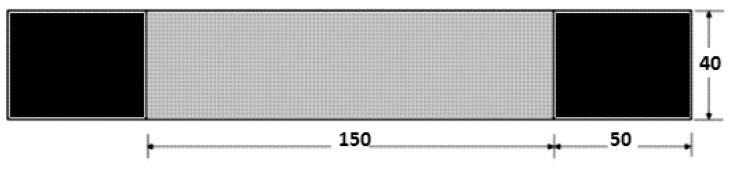
Tensile test specimen geometry (dimensions in mm).

Tensile tests were performed on specimens of glass fiber-reinforced polymer. These tests were performed using a computer-controlled universal electromechanical testing machine (machine model WDW-100- Jinan Victory Instrument Co. Ltd., Jinan, China) [40], with a load capacity of 100 kN and a controlled speed of 2 mm/min, in accordance with ASTM D3039 [41], for tension. The typical specimen geometry for tension is shown in Figure 4.

### 3.3. Drop Weight Impact Test

Due to their high specific strength and modulus, low specific density, and corrosion resistance, fiber-reinforced polymers are widely used in aerospace, transportation, and construction applications. In order to achieve an optimal design of such a material, standard testing and determinations of mechanical properties are required. Toughness testing, like impact testing, is an important test of fiber-reinforced plastics that is used to determine the material’s ability to absorb energy. Although Izod and Charpy are the best known impact testing methods, they have significant drawbacks, such as the need for a notch in the specimen and limitations on the amount of load applied. For impact testing, another approach, drop weight impact testing (DWIT) [42], can be used. The energy absorption capacity of materials is measured by dropping a weight onto the specimen. Compared to metals, composites show different responses when subjected to impact loading. While metals show a rapid elastic response followed by protracted plastic deformation under impact loads, composites show an elastic response followed by a variety of failure modes such as delamination, cracking of the matrix, and fiber breakage. This could be due to the fact that the impact energy in metals is absorbed by plastic deformation, while the energy in composites is absorbed by different failure modes. 

**Figure 5:**
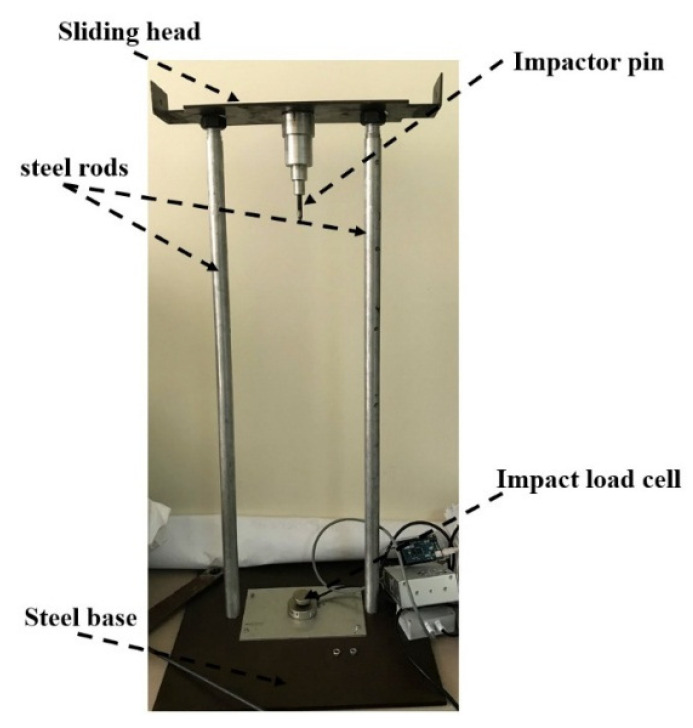
Simple drop weight impact tester.

Figure 5 shows the test fixture. It consists of two 1000 mm long steel bars bolted to a rigid steel plate, with the upper ends of the bars restrained by a steel beam. A cross steel head connects the impactor to the two steel bars. The transverse steel head was designed to move over the two rods with the impactor, allowing the surfaces of the specimens to fall freely. The depth of the indentation caused by the penetration of the pin into the surface of the specimen is measured. The absorption of energy by the material is measured using the depth of penetration. The law of conservation of energy is used to calculate the velocity of the falling load and the impact time. The specimens were square, with an edge length of 30 mm made of the two materials under study, fiberglass cloth and carbon fiber with eight layers. The specimens were simply supported at the edge on the impact load cell, sinve clamping is not preferred, especially for ductile material, to prevent the buckling of the outer region of the specimen [43] For each load of 0.5 and 1 kg, three different heights were used: 0.5, 1 and 1.5 m.

### 3.4. Relaxation Test

Stress relaxation is defined as a noticeable reduction in tension in response to stress on the structure. This is because a structure held in a stressed state for an extended period of time will exhibit some plastic strain. Therefore, this concept is not inconsistent with creep, where increasing strain is associated with a continuous state of stress. Since relaxation causes a significant reduction in the stress level, it is reflected in the reduction in equipment response. Relaxation differs from cold spinning in that it occurs over a longer period of time; however, both have the same effect. The amount of relaxation that occurs depends on several variables, such as time, temperature, and load level. Therefore, the exact effect on the system is unknown, although it can be limited. When stress is sustained, stress relaxation shows how polymers can provide stress relief. Since polymers are viscoelastic and not subject to Hooke’s law, they behave nonlinearly [44]. Stress relaxation and a phenomenon known as creep define the aforementioned nonlinearity, which shows how polymers stretch under constant stress. At any point in the course of a constant strain rate or a creep test, a relaxation test can be performed. Ideally, the length of the specimen should be kept constant throughout. This will dissipate the stored elastic strain energy of the specimen through plastic deformation, resulting in a decrease in the determined value of the stress maintained by the specimen over time [45].

**Figure 6:**
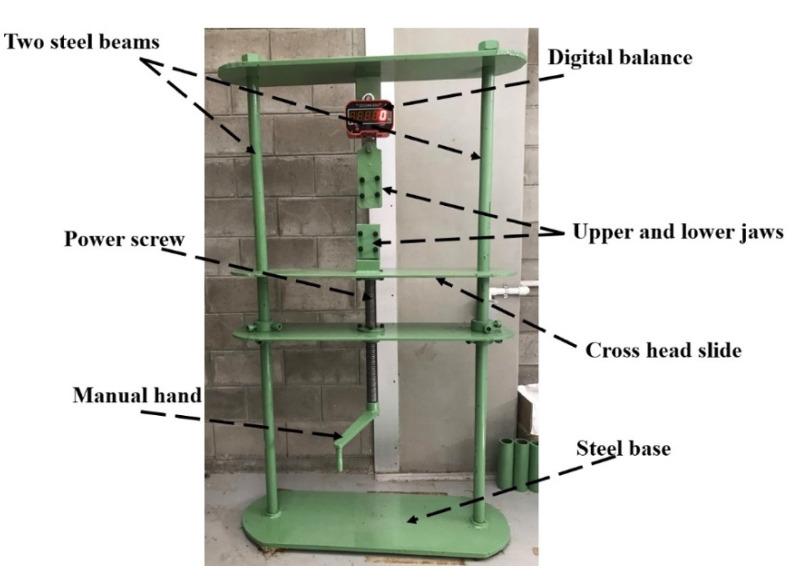
Manual relaxation testing device.

A simple digital spring balance with a maximum capacity of 50 kN was built to evaluate the relaxation response of such a material. It was coupled with robust steel beams. As shown in Figure 2, one end of a conventional flat tensile specimen was fixed in this balance with a designed coarse upper frame handle, while the other end was clamped to the second lower frame handle attached to the mechanical manual force screw (see Figure 6). To increase the gripping force at the ends of the specimen and prevent slippage from the rough handles, both ends are wrapped with emery paper. 

The load is transferred to the specimen via a force screw until the balance reaches the prescribed load, which corresponds to the loads on the force–displacement curve of the basic tensile test. For all specimens measured, the load was set at 40 percent of the maximum load of the lowest strength to ensure that the higher strength specimens were safe, as measured by a basic tensile test.

The relaxation young modulus can be measured as follows:

(1) $$E_{r}=\frac{\sigma \left(t\right)}{\epsilon \left(t\right)}$$

**Figure 7:**
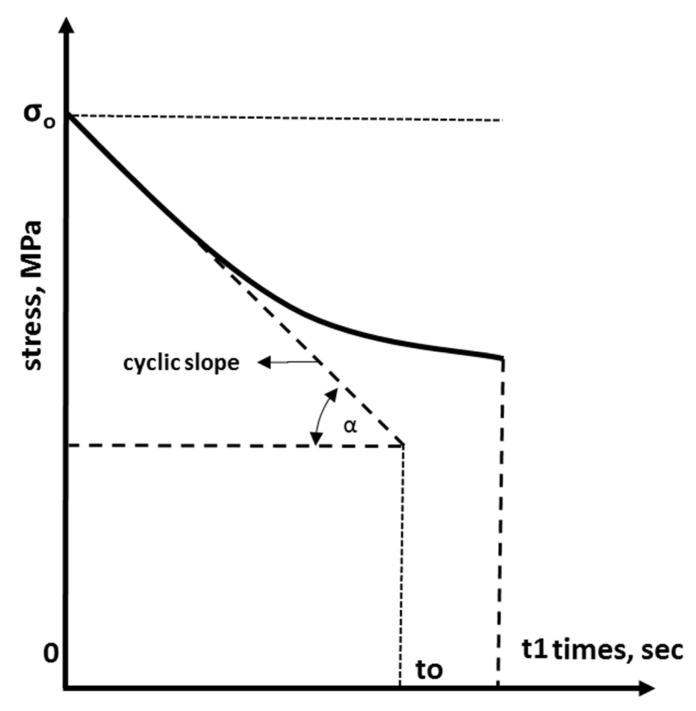
Experimental cyclic slope.

The cyclic slope (*α*) can be measured from the stress time curves, as shown in Figure 7. The slope of the curve *α* can be measured as follows:

(2) $$\mathrm{Cyclic}\;\mathrm{slope}=\alpha =\frac{\sigma_{0}}{t_{o}}\;\mathrm{kW}/m^{3}\;$$

## 4. Center Notch Specimen

The evaluation of fracture toughness is still very important in the application of composite laminates. This is because fracture toughness is a measure of a material’s ability to resist crack propagation. The center-cracked tension (CCT) specimen is a popular choice.

To calculate the surface release energy of such hybrid composites, Soutis et al. created a model [46] using quasi-brittle fabric laminates with center-cracked tension plate specimens. The cured plates were first cut to their nominal dimensions using a diamond-coated disk. After the specimens were machined to their final geometry, a pre-crack length of 10 mm was applied.

**Figure 8:**
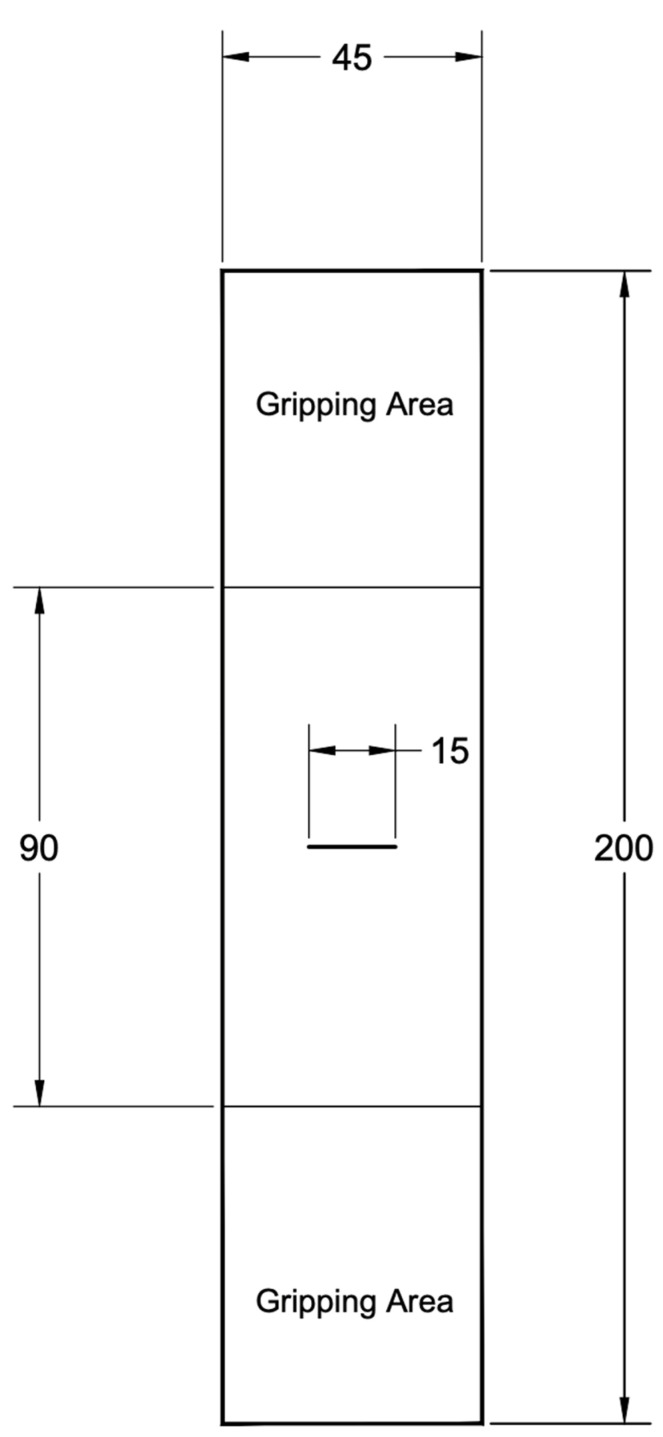
Center-cracked tension (CCT) specimen geometry (dimensions in mm) [32].

The geometry of the CCT specimens used is shown in Figure 8. For each case, five specimens with a length of 15 mm were prepared for the central crack (2a). The specimens were then subjected to tensile loading until they failed, with the load and displacement being recorded.

## 5. Results and Discussion 

### 5.1. Tension Test

**Figure 9:**
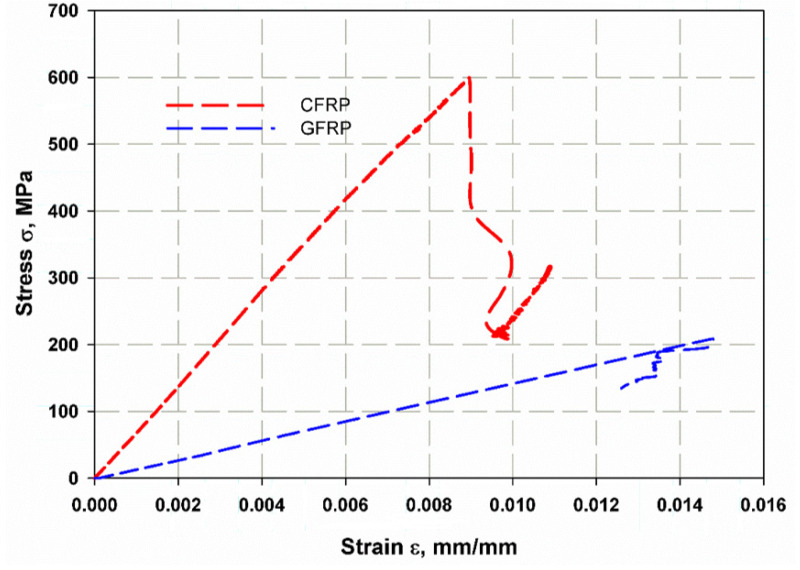
Stress versus strain ratio of tensile specimens.

**Figure 10:**
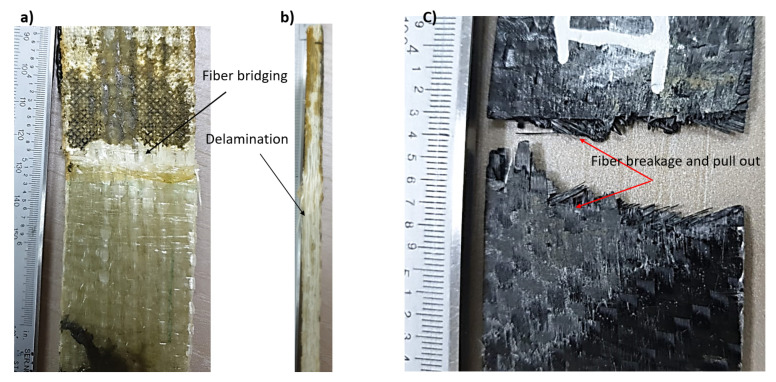
Failure modes in tension tests of specimens reinforced by; (**a**) glass fiber, (**b**) delamination (**c**) carbon fiber.

Figure 9 shows the relationship between stress and strain for the carbon fiber (red line) and glass fiber (blue line) reinforced epoxy laminates. Both materials behave elastically and linearly, but for the carbon fiber- reinforced material, failure is delayed for a while after the peak stress is reached and rapid failure begins, because the fibers still form a bridge and resist failure. The carbon fiber-reinforced specimens exhibited higher strength than the glass fiber-reinforced ones, with values of 600 MPa for the carbon fiber-reinforced epoxy laminates and 200 MPa for the glass fiber-reinforced laminates, which was due to the high strength and stiffness of the carbon fibers and the very high attachement with the ascended epoxy interfaces as compared to epoxy with glass fibers. This effect can be seen in Figure 10, as the fracture surface of the composite laminates reinforced with carbon fibers was not straight but was roughly graded on the light side (see Figure 10b), while the fracture surface of the specimens reinforced with glass fibers with greater thickness was almost straight (see Figure 10a). Moreover, severe delamination was observed throughout the thickness of the specimen, while this was not the case for the thin carbon fiber-reinforced specimens. This may explain the reason for the high strength of carbon fiber-reinforced epoxy laminates compared to glass fiber-reinforced laminates, apart from the higher specific strength and stiffness compared to glass fibers. The Young’s modulus was measured from the stress-strain diagram (Figure 9) as (68.7 and 29.5) GPa for CFRP and GFRP, respectively. 

### 5.2. Center Crack Notch

**Figure 11:**
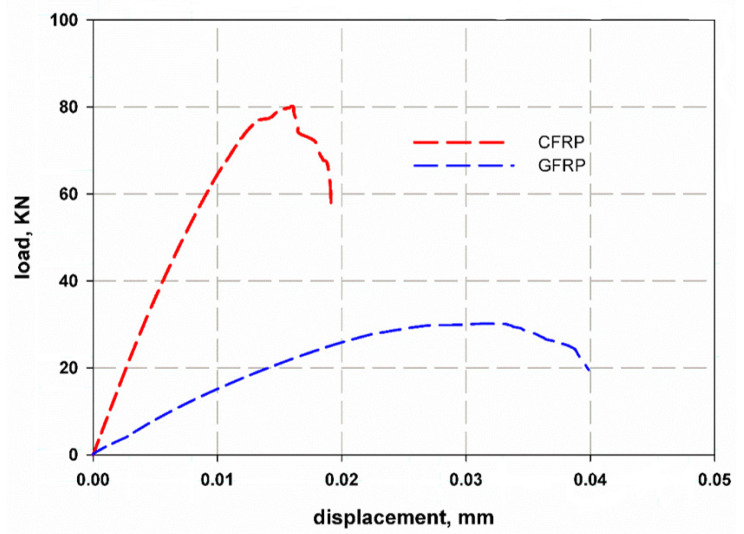
Load versus displacement relationship for center crack notch specimen.

**Figure 12:**
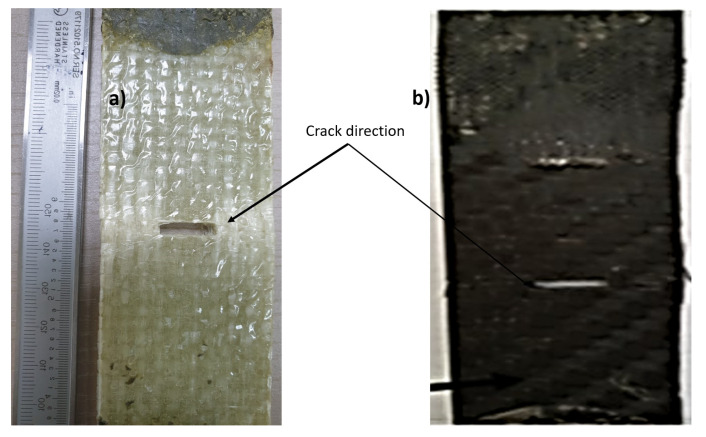
Failure modes for the center crack notch specimens; (**a**) glass fiber, (**b**) carbon fiber.

Figure 11 shows the relationship between load and displacement for the mean crack notch. The average maximum loads measured are 80 kN and 25 kN for carbon and glass fiber-reinforced epoxy laminates, respectively. The average failure stress is 889 MPa and 138.8 MPa for carbon fiber (CFRP) and glass fiber-reinforced (GFRP) epoxy composite laminates, respectively. The loading behavior is uniform with a flat failure plateau for glass fiber-reinforced epoxy laminates. The failure modes are net stress modes with fiber bridging for both specimens (see Figure 12). The energy released at the fracture surface can be measured using Equation (3) considering the failure stress for each specimen and using the real dimensions of the specimen as follows [32]:

(3) $$K_{Ic}=\sigma \sqrt{\pi a}\sqrt{sec\left(\frac{\pi a}{w}\right)}$$

where *σ* is the fracture stress, *a* is half the crack length, and *w* is the specimen width. The average fracture toughness (*K_Ic_*) was measured as (192, 31) MPa√m for CFRP and GFRP, respectively. The average surface release energy *G_IC_* was measured as (540.6, 31.1) kJ/m^2^ for CFRP and GFRP, respectively. The large differences between the values of CFRP and GFRP can be attributed to the large difference in the stiffness and strength of carbon fibers compared to glass fibers. The large thickness of the laminates of glass fiber composites results in delamination through the thickness, while the cracks in the carbon fibers composites were almost like a tensile test, with two cracks on the two sides of the specimens. It was found that the maximum elongation reached 0.04 mm for GFRP, while it was 0.02 mm for CFRP. This was due to the greater thickness and amount of elastic epoxy in the glass fiber layers, where the epoxy volume fraction was 55%, while it was 35% for the carbon fibers. The volume fraction was measured using the ignition technique, according to the ASTM D3171-99 standard [39].

### 5.3. Relaxation Test

**Figure 13:**
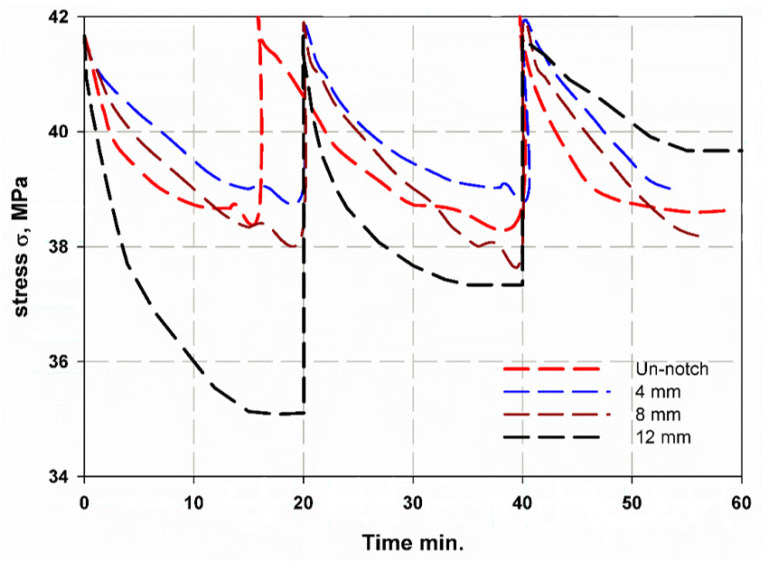
Relaxation stress and time curve of CFRP.

**Figure 14:**
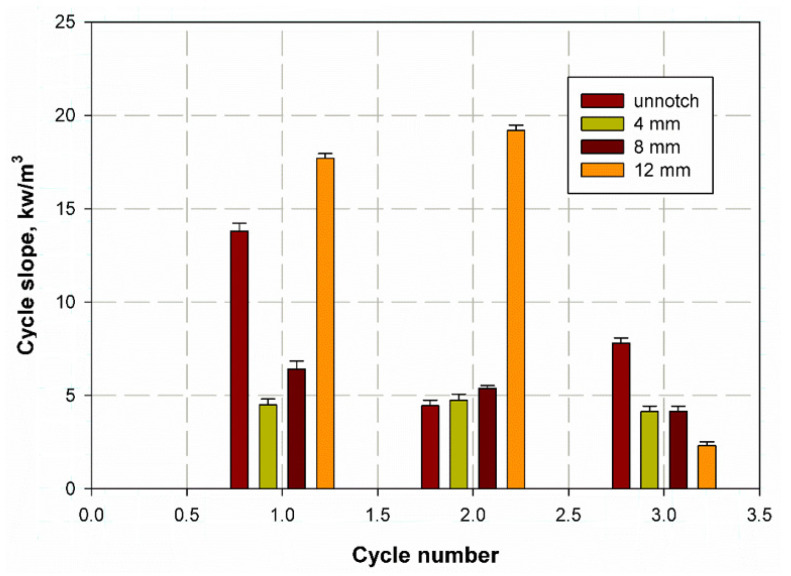
Cycle slope versus cycle number of CFRP.

**Figure 15:**
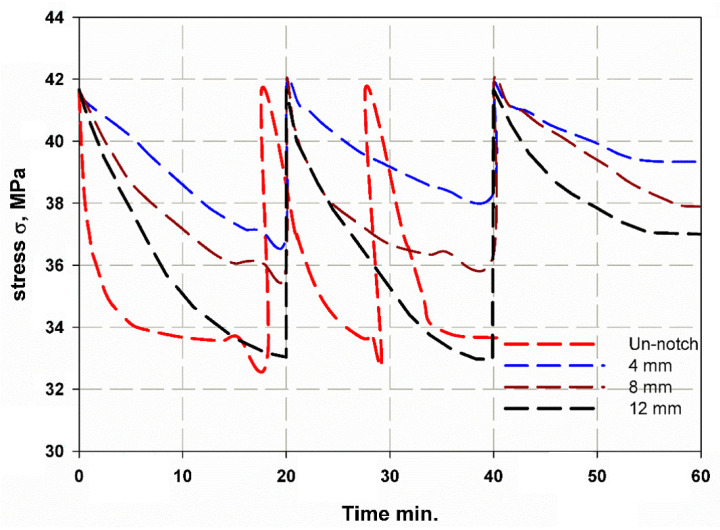
Relaxation stress and time curve of GFRP.

**Figure 16:**
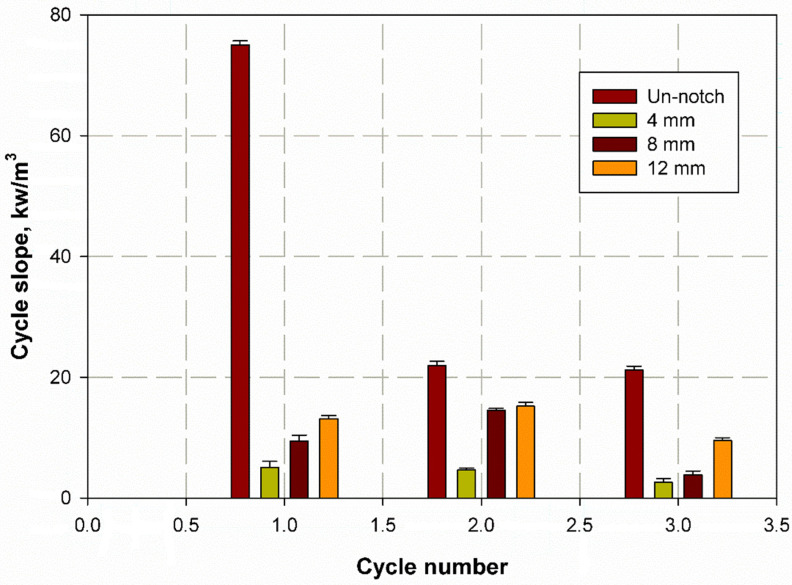
Cycle slope versus cycle number of GFRP.

**Figure 17:**
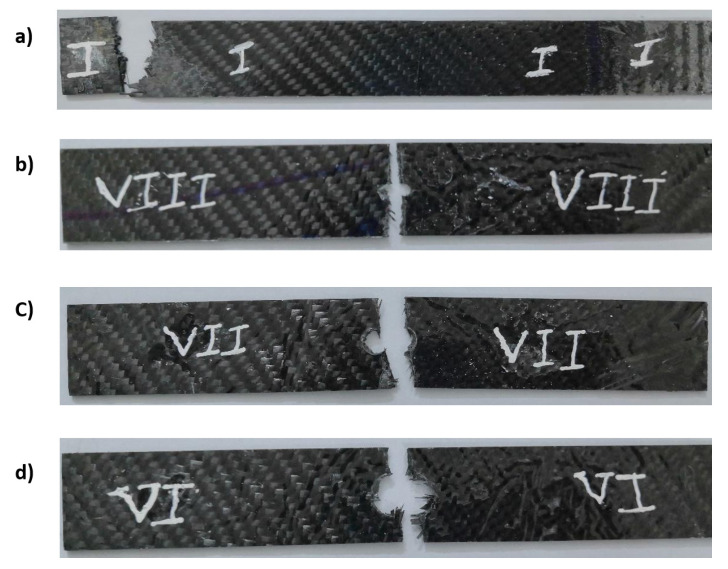
Failure modes in the relaxation test for CFRP with hole diameters (**a**) no notch, (**b**) 4 mm, (**c**) 8 mm, (**d**) 12 mm.

**Figure 18:**
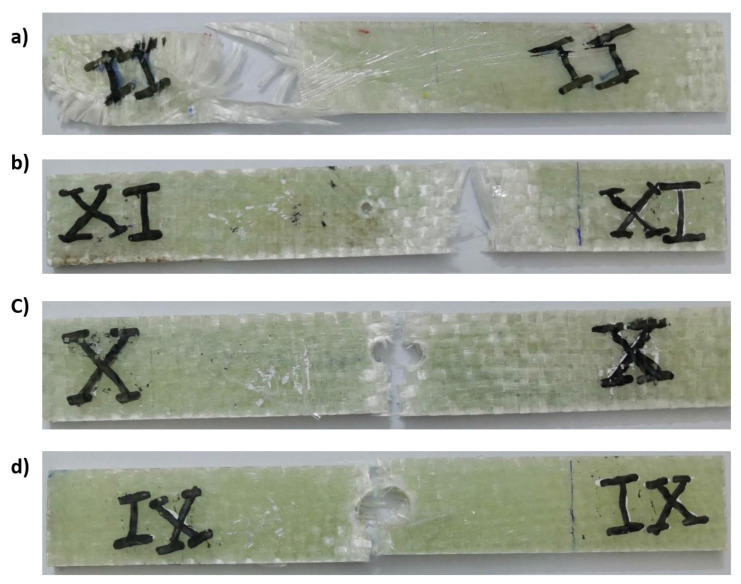
Failure modes in the relaxation test for GFRP with hole diameters (**a**) no notch, (**b**) 4 mm, (**c**) 8 mm, (**d**) 12 mm.

Figure 13 shows the stress as a function of time for CFRP. It can be observed that the diameter of the hole affects the curve trend. For the sample with increasing hole diameter of 12 mm, the stress was high and had a steep slope, as shown in Figure 13. The number of cycles was almost three, where the first and second cycles had the same duration of 20 min, while the last cycles were shorter. Towards the end of the first and second cycles, a wave kink was observed, which was due to the softening of the epoxy resin. For the first and second cycles, the cyclic slope increased with the hole diameter (see Figure 14). This was due to the decrease in strength with increasing hole diameter, leading to rapid failure, while the trend changed for the last few cycles as the epoxy matrix around the holes cracked. It was also important to observe that the lowest stress shifts with each cycle. This can be attributed to the fact that the load carrying capacity decreased as the cycle time increased. For GFRP, the same trend can be seen in Figure 15, but the first and second cycles have almost the same minimum stress, and the slope of the last cycle (see Figure 16) was also different from that of CFRP. For both materials, CFRP and GFRP, the unnotched specimens show an increasing cyclic slope, which was due to the high strength of the carbon and glass fibers, which increased the stress concentration near the machine gaps, and also to the changes in the stress distribution during manual testing. All failure modes were net stresses with coarse cracks on the surface. No delaminations were observed in the CFRP, while slight delaminations were observed in the GFRP. The failure mode in the case of unnotched GFRP was tearing, as the surfaces were destroyed (Figure 17 and Figure 18).

### 5.4. Drop Weight Impact Test

**Figure 19:**
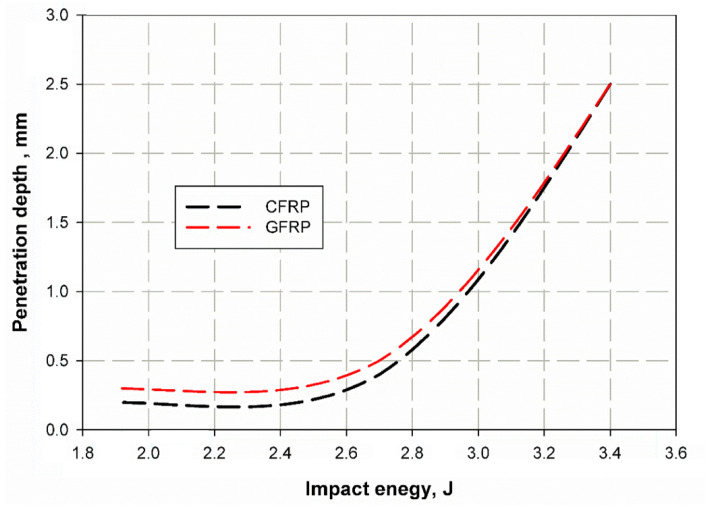
Depth of indentations in the drop weight impact test.

**Figure 20:**
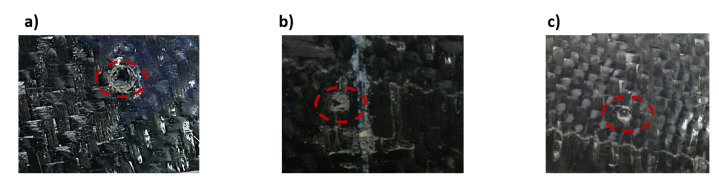
Tndentation though CFRP for (**a**) 1.9 J, (**b**) 2.7 J, and (**c**) 3.4 J.

**Figure 21:**
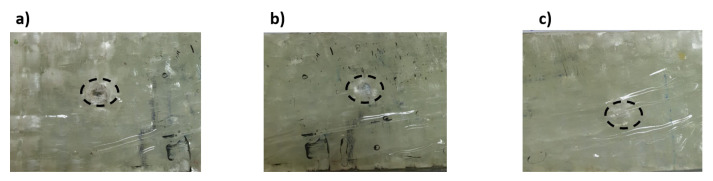
Indentation though GFRP for (**a**) 1.9 J, (**b**) 2.7 J, and (**c**) 3.4 J.

The drop weight impact test is illustrated in Figure 19 for both CFRP and GFRP. It can be observed that the indentations in the specimens reinforced with glass fiber composite laminates were larger than those of the specimens reinforced with carbon fibers in the range of impact energy from 1.9 to 2.7 J. This difference decreased in the range of impact energy from 2.7 to 3.4 J, which was due to the fact that, in this range, the impactor penetrates deep into the material and hits both the carbon and glass fibers, which have a high impact force. The indentations for all specimens are shown in Figure 20 and Figure 21 for the CFRP and GFRP, respectively. The depth of the indentation through the thickness increased with increasing velocity, which was due to an increase in the kinetic energy of the impact (𝑒 = 1/2 𝑚𝑣^2^), some of which was stored in the specimen in the form of crack depth, while the rest is dissipated was the form of impact noise and temperature. Depth increases more readily than velocity with increasing load, which may be due to the fact that energy changes only once with velocity. The depth of the indentation can be considered a measure of the energy stored in the material. The elastic response of metal under impact loading becomes short while the plastic deformation is long. On the other hand, the elastic response of composite materials results in softening, with various forms of failure such as delamination, bridging and cracking in the matrix. Therefore, the absorption of energy in metal is dissipated by plastic deformation, while in composites the energy has been observed in many failure modes.

## 6. Concussions

Composite laminates have many excellent and competitive properties. The tensile strength of CFRP and GFRP were measured, these two values were selected to determine the 40% relaxation stress for each sample. The relaxation behavior and cyclic slope were measured. It was found that both CFRP and GFRP with holes exhibited the same three relaxation cycles at the same percent stress, but with changing behavioral trends. The cyclic slope of relaxation increases with increasing hole diameter, but for composite specimens without notched, it decreases with time for both types of material. Glass fibers show better impact behavior than carbon fibers at impact energies of 1.9 J to 2.7 J, while the impact behavior was more similar at impact energies of 2.7 J to 3.4 J. The fracture toughness KIC was measured to be 192 MPa √m and 31 MPa √m, and the surface energy GIC was measured to be 540.6 kJ/m^2^ and 31.1 kJ/m^2^ for carbon and glass fiber-reinforced epoxy laminates, respectively.

## Figures and Tables

**Table 1:** Mechanical and physical properties of E-glass fiber and epoxy resin [32,37,38].

Properties	E-Glass	Kemapoxy (150RGL)
Density (kg/m^3^)	2600	1.2
Tensile strength (MPa)	3450	85
Tensile modulus (GPa)	80	2.5
Passion ratio	0.25	0.35
In plane shear modulus	30.8	1.24

## Data Availability

The data presented in this study are available on request from the corresponding author.

## References

[1] Abdellah M.Y., Alsoufi M.S., Hassan M.K., Ghulman H.A., Mohamed A.F. (2015). Extended finite element numerical analysis of scale effect in notched glass fiber reinforced epoxy composite. Arch. Mech. Eng..

[2] Alharthi H., Abdellah M.Y., Abdo H.S., Hassan M.K. (2020). Size Effect and Fracture Toughness of Glass Fiber Composite Structures. Int. J. Adv. Sci. Technol..

[3] Mohammed Y., Hassan M.K., El-Ainin H.A., Hashem A.M. (2013). Size Effect Analysis in Laminated Composite Structure using General Bilinear Fit. Int. J. Nonlinear Sci. Numer. Simul..

[4] Mohammed Y., Hassan M.K., El-Ainin H.A., Hashem A.M. (2015). Size effect analysis of open-hole glass fiber composite laminate using two-parameter cohesive laws. Acta Mech..

[5] Guo R., Xian G., Li C., Huang X., Xin M. (2021). Effect of fiber hybridization types on the mechanical properties of carbon/glass fiber reinforced polymer composite rod. Mech. Adv. Mater. Struct..

[6] Xian G., Guo R., Li C., Hong B. (2021). Effects of rod size and fiber hybrid mode on the interface shear strength of carbon/glass fiber composite rods exposed to freezing-thawing and outdoor environments. J. Mater. Res. Technol..

[7] Abdellah M.Y., Sulaiman S., Mohamed A.F., Gomaa A.A., Abdel-Jaber G.T. (2019). Relaxation, Impact and Flexural Properties of Glass Fiber Reinforced Epoxy. Int. J. Mech. Mechatron. Eng. IJMME-IJENS.

[8] Abdellah M.Y., Mohamed A.F., Hasan M.K. (2019). Characteristic Analysis: Vibration Behaviour of Composite Laminated Structures Compared to Monotonic Materials. Int. J. Mech. Mechatron. Eng. IJMME-IJENS.

[9] Fouad H., Mourad A.H.I., ALshammari B.A., Hassan M.K., Abdallah M.Y., Hashem M. (2020). Fracture toughness, vibration modal analysis and viscoelastic behavior of Kevlar, glass, and carbon fiber/epoxy composites for dental-post applications. J. Mech. Behav. Biomed. Mater..

[10] Mohammed Y., Hassan M.K., Hashem A. (2014). Analytical model to predict multiaxial laminate fracture toughness from 0 ply fracture toughness. Polym. Eng. Sci..

[11] Gong Y., Zhao L., Zhang J., Hu N., Zhang C. (2021). Development of a standardized test procedure and an improved data reduction method for the mixed-mode I/II delamination in composite laminates. Compos. Sci. Technol..

[12] Ouyang T., Sun W., Bao R., Tan R. (2021). Effects of matrix cracks on delamination of composite laminates subjected to low-velocity impact. Compos. Struct..

[13] Staniszewski J.M., Boyd S.E., Bogetti T.A. (2022). A multi-scale modeling approach for UHMWPE composite laminates with application to low-velocity impact loading. Int. J. Impact Eng..

[14] Papanicolaou G., Zaoutsos S. (2019). Viscoelastic constitutive modeling of creep and stress relaxation in polymers and polymer matrix composites. Creep and Fatigue in Polymer Matrix Composites.

[15] Eftekhari M., Fatemi A. (2016). On the strengthening effect of increasing cycling frequency on fatigue behavior of some polymers and their composites: Experiments and modeling. Int. J. Fatigue.

[16] Eftekhari M., Fatemi A. (2016). Creep behavior and modeling of neat, talc-filled, and short glass fiber reinforced thermoplastics. Compos. Part B Eng..

[17] Obaid N., Kortschot M.T., Sain M. (2017). Understanding the stress relaxation behavior of polymers reinforced with short elastic fibers. Materials.

[18] George J., Sreekala M.S., Thomas S., Bhagawan S.S., Neelakantan N.R. (1998). Stress relaxation behavior of short pineapple fiber reinforced polyethylene composites. J. Reinf. Plast. Compos..

[19] Pothan L.A., Neelakantan N.R., Rao B., Thomas S. (2004). Stress relaxation behavior of banana fiber-reinforced polyester composites. J. Reinf. Plast. Compos..

[20] Obaid N., Kortschot M.T., Sain M. (2017). Modeling and predicting the stress relaxation of composites with short and randomly oriented fibers. Materials.

[21] Saeed U., Al-Turaif H., Siddiqui M.E. (2021). Stress relaxation performance of glass fiber-reinforced high-density polyethylene composite. Polym. Polym. Compos..

[22] Arif M., Saintier N., Meraghni F., Fitoussi J., Chemisky Y., Robert G. (2014). Multiscale fatigue damage characterization in short glass fiber reinforced polyamide-66. Compos. Part B Eng..

[23] Sato N., Kurauchi T., Sato S., Kamigaito O. (1991). Microfailure behaviour of randomly dispersed short fibre reinforced thermoplastic composites obtained by direct SEM observation. J. Mater. Sci..

[24] Pérez-Pacheco E., Moreno-Chulim M.V., Valadez-González A., Rios-Soberanis C.R., Herrera-Franco P.J. (2011). Effect of the interphase microstructure on the behavior of carbon fiber/epoxy resin model composite in a thermal environment. J. Mater. Sci..

[25] Ma L., Meng L., Wu G., Wang Y., Zhao M., Zhang C., Huang Y. (2015). Effects of bonding types of carbon fibers with branched polyethyleneimine on the interfacial microstructure and mechanical properties of carbon fiber/epoxy resin composites. Compos. Sci. Technol..

[26] Wu G., Ma L., Liu L., Wang Y., Xie F., Zhong Z., Zhao M., Jiang B., Huang Y. (2015). Interfacially reinforced methylphenylsilicone resin composites by chemically grafting multiwall carbon nanotubes onto carbon fibers. Compos. Part B Eng..

[27] Varghese S., Kuriakose B., Thomas S. (1994). Stress relaxation in short sisal-fiber-reinforced natural rubber composites. J. Appl. Polym. Sci..

[28] Ahmed F.M., Mohammed Y.A., Mohammed K.H. (2015). Relaxation and Compressive Characteristic in Composite Glass Fiber reinforced Pipes. Int. J. Sci. Eng. Res..

[29] Ornaghi H.L., Almeida J.H.S., Monticeli F.M., Neves R.M. (2020). Stress relaxation, creep, and recovery of carbon fiber non-crimp fabric composites. Compos. Part C Open Access.

[30] Tobushi H., Matsui R., Takeda K., Hayashi S. (2015). Mechanical testing of shape-memory polymers for biomedical applications. Shape Memory Polymers for Biomedical Applications.

[31] Khashaba U.A. (2004). In-plane shear properties of cross-ply composite laminates with different off-axis angles. Compos. Struct..

[32] Mohammed Y., Hassan M.K., Hashem A.M. (2014). Effect of stacking sequence and geometric scaling on the brittleness number of glass fiber composite laminate with stress raiser. Sci. Eng. Compos. Mater..

[33] Hassan M.K., Abdellah M.Y., Azabi S.K., Marzouk W.W. (2015). Fracture Toughness of a Novel GLARE Composite Material. Int. J. Eng. Technol. IJET-IJENS.

[34] Korim N.S., Abdellah M.Y., Dewidar M., Abdelhaleem A.M. (2015). Crushable Finite Element Modeling of Mechanical Properties of Titanium Foam. Int. J. Sci. Eng. Res..

[35] Mohamed K., Hassan Y.M., Abu El-Ainin H. (2012). Improvement of Al-6061 alloys mechanical properties by controlling processing parameters. Int. J. Mech. Mechatron. Eng. IJMME-IJENS.

[36] Hassan M.K., Abdellah M.Y., Azabi S.K., Marzouk W. (2015). Investigation of the Mechanical Behavior of Novel Fiber Metal Laminates. Int. J. Mech. Mechatron. Eng. IJMME-IJENS.

[37] Hagnestål A., Sellgren U., Andersson K. Durable winch-based point absorbers. Proceedings of the Ewtec 2017: The 12th European Wave and Tidal Energy Conference.

[38] Shukla M.J., Kumar D.S., Mahato K.K., Rathore D., Prusty R.K., Ray B.C. (2015). A comparative study of the mechanical performance of Glass and Glass/Carbon hybrid polymer composites at different temperature environments. IOP Conf. Ser. Mater. Sci. Eng..

[39] ASTM D3171 (2011). Standard Test Methods for Constituent Content of Composite Materials.

[40] http://www.victorytest.com/products/wdw-50100-and-computerized-electromechanical-universal-testing-machine.

[41] ASTM D3039/D 3039M (1995). Standard Test Method for Tensile Properties of Polymer Matrix Composite Materials.

[42] Taheri-Behrooz F., Shokrieh M.M., Abdolvand H. (2013). Designing and manufacturing of a drop weight impact test machine. Eng. Solid Mech..

[43] Swallowe G.M. (1999). Mechanical Properties and Testing of Polymers: An A–Z reference.

[44] Meyers M.A., Chawla K.K. (2008). Mechanical Behavior of Materials.

[45] Rutter E., Atkinson B., Mainprice D. (1978). On the use of the stress relaxation testing method in studies of the mechanical behaviour of geological materials. Geophys. J. Int..

[46] Soutis C., Curtis P.T., Fleck N.A. (1993). Compressive failure of notched carbon fibre composites. Proc. R. Soc. Lond. Ser. A Math. Phys. Sci..

